# 1397. Modern Lineages of *Mycobacterium tuberculosis* Were Recently Introduced in Western India and Demonstrate Increased Transmissibility

**DOI:** 10.1093/ofid/ofab466.1589

**Published:** 2021-12-04

**Authors:** Avika Dixit, Anju Kagal, Yasha Ektefaie, Luca Freschi, Rahul Lokhande, Matthias Groeschel, Jeffrey A Tornheim, Nikhil Gupte, Neeta N Pradhan, Deelip Kadam, Amita Gupta, Jonathan Golub, Maha Farhat, Vidya Mave

**Affiliations:** 1 Boston Children’s Hospital, Harvard Medical School, Boston, Massachusetts; 2 Byramjee-Jeejeebhoy Medical College-Johns Hopkins University Clinical Research Site, Pune, India, Pune, Maharashtra, India; 3 Harvard Medical School, Boston, Massachusetts; 4 Johns Hopkins University School of Medicine, Baltimore, Maryland; 5 Johns Hopkins University, Pune, Maharashtra, India; 6 JHU-BJMC CTU, Pune, Maharashtra, India; 7 Johns Hopkins, Baltimore, MD

## Abstract

**Background:**

*Mycobacterium tuberculosis* (*Mtb*) transmissibility may vary between lineages (or variants) and this may contribute to the slow decline of tuberculosis incidence. The objective of our study was to compare transmissibility across four major lineages (L1-4) of *Mtb* in Pune, India.

**Methods:**

We performed whole-genome sequencing (WGS) of *Mtb* isolated from sputum culture of adult patients with pulmonary TB. We performed genotypic susceptibility testing for both first- and second-line drugs using a previously validated random forest predictor. We identified single nucleotide polymorphisms and generated a multiple sequence alignment excluding drug resistance conferring mutations to avoid skewing the phylogeny due to convergent evolution in these regions. We used Bayesian molecular dating to generate phylogenies and compared tree characteristics using a two-sample Kolmogorov-Smirnov (KS) test.

**Results:**

Of the 642 isolates from distinct study participants that underwent WGS, 612 met quality criteria. The median age of participants was 31 years (range 18-74), the majority were male (64.7%) and sputum smear-positive (83.3%), and 6.7% had co-infection with HIV (Table 1). There was no significant difference in baseline characteristics between lineages. The majority of isolates belonged to L3 (44.6%). The majority (61.1%) of multidrug-resistant (MDR, resistant to isoniazid and rifampin) isolates belonged to L2. In phylogenetic analysis, we found evidence of higher transmissibility of L2 as indicated by shorter branch lengths (i.e., less time had elapsed between transmission and sampling) and more genetic similarity (smaller pairwise single nucleotide polymorphism [SNP] distances) among L2 isolates as compared to other lineages (Figure 1). Branching times for L2 and L4 were smaller than L1 and L3 indicating recent introduction into the region (p < 0.001 [KS test]).

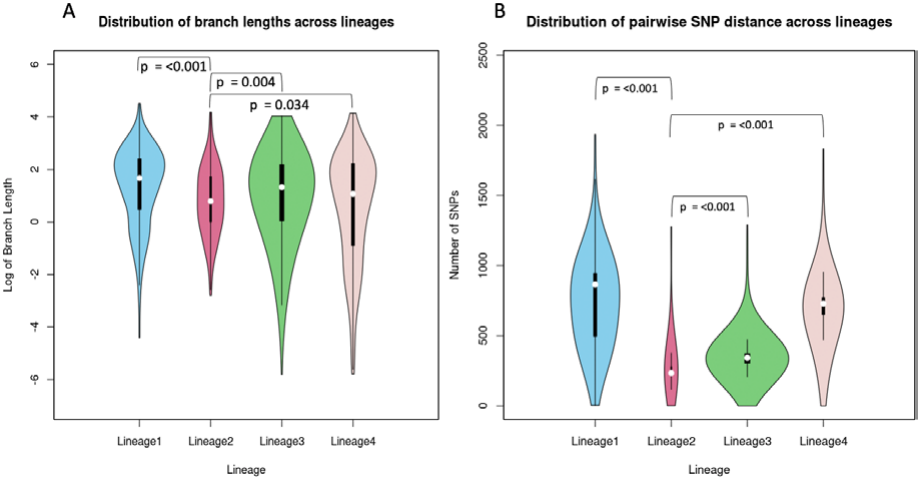

Figure 1: Lineage-wise distribution of A) phylogenetic tree branch lengths (log) and B) pairwise single nucleotide polymorphism (SNP) distance, using 612 tuberculosis isolates from Pune, India. P values calculated using two-sample Kolmogorov-Smirnov test.

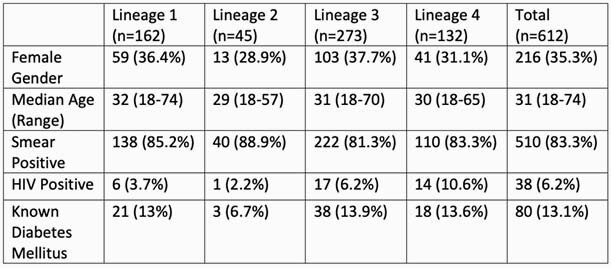

Table 1: Demographic characteristics of study participants included in the study, by lineage.

**Conclusion:**

Modern *Mtb* lineages (L2 and L4) were relatively recently introduced in western India, as compared to older lineages (L1 and L3), with the more drug-resistant L2 showing higher transmissibility. These findings highlight the need for early detection and treatment initiation to interrupt transmission with important implications for antimicrobial stewardship and heightened surveillance of TB resistance rates.

**Disclosures:**

**All Authors**: No reported disclosures

